# Right ventricular dysplasia in the elderly: a case report from autopsy

**DOI:** 10.11604/pamj.2021.38.404.29250

**Published:** 2021-04-28

**Authors:** Alban Ikenna Mgbehoma, Oluwaseye Olumide Onayemi, Sunday Sokunle Soyemi, John Oladapo Obafunwa

**Affiliations:** 1 Department of Pathology and Forensic Medicine, Lagos State University Teaching Hospital, Ikeja, Lagos, Nigeria

**Keywords:** Right ventricular cardiomyopathy, right ventricular dysplasia, case report

## Abstract

Right ventricular dysplasia (RVD) is a rare disease of the heart that primarily affects the right ventricle. It is a clinical and pathological entity that presents classically with palpitations, syncope, or even sudden death. It presents rarely in the elderly. Where sudden death is the first and only presentation, an autopsy is required to make the diagnosis. However, the pathomorphological features of RVD can easily be overlooked or missed at autopsy. We report the case of a 68-year-old male with the past medical history of hypertension, gout and inflammatory bowel syndrome. He was admitted on account of difficulty in breathing, abdominal swelling and reduced urination. Physical examination revealed hypertension with cardiac murmurs, widespread crepitations, distended abdomen and lower limb oedema. Provisional diagnoses of acute-on-chronic kidney disease and congestive cardiac failure secondary to hypertensive heart disease, precipitated by probable gastrointestinal infection were made. While on admission, he had an episode of syncope. Electrocardiogram revealed bigeminy and bradycardic sinus rhythm with unifocal ventricular premature contraction. He died on the 8^th^ week of admission. Autopsy revealed an enlarged heart weighing 600gm; there was thinning of the apical aspect of the right ventricular wall with subtotal fibrofatty replacement. Microscopic examination revealed a transmural replacement of cardiac myocytes by fibroadipose tissue extending inwards, in the most parts, from the epicardium to the endocardial surface. Our aim is to increase the awareness of these pathomorphological features among anatomic/forensic pathologists.

## Introduction

Right ventricular dysplasia (RVD) is a rare disease of the heart that primarily affects the right ventricular musculature [1]. This disease may impair the pump function of the heart or cause irregular heartbeats and these disturbances of normal heart function may culminate in sudden death [2]. It has been implicated as a cause of sudden death in the young, particularly athletes [3,4] and rarely manifests in the elderly [5]. Where sudden death is the clinical presentation, diagnosis can only be made if post mortem examination is conducted [6]. Making such a diagnosis at autopsy requires an established and functional medico-legal system that makes sudden and unexpected death a reportable case. However, this diagnosis can be easily missed or overlooked by the anatomic/forensic pathologists [7].

We report the case of a now deceased elderly male patient who had right ventricular dysplasia. A search of the Nigerian literature did not reveal any previous documentation of RVD. This report describes the pathomorphological features of RVD and reviews the literature. Our aim is to increase the awareness of these features among anatomic/forensic pathologists.

## Patient and observation

The now deceased was a 68-year-old male with a past medical history of hypertension, gout and inflammatory bowel syndrome. He was admitted on account of difficulty in breathing, abdominal swelling and reduced urination. Physical examination revealed hypertension with cardiac murmurs, widespread crepitations, distended abdomen and lower limb oedema. Provisional diagnoses of acute-on-chronic kidney disease and congestive cardiac failure secondary to hypertensive heart disease, precipitated by probable gastrointestinal infection were made. Laboratory evaluation showed azotaemia, hyponatraemia, hyperkalaemia, hyperphosphataemia, hyperuricaemia, elevated creatinine and elevated erythrocyte sedimentation rate (ESR). He was urgently dialysed. While on admission, he was managed for diarrhoea, bladder outlet obstruction and gouty arthritis; however, on the 20^th^ day of admission, he had an episode of syncope. Following the syncopal episode, an electrocardiogram (ECG) was done and it revealed bigeminy and bradycardic sinus rhythm with unifocal ventricular premature contraction.

**Figure 1:**
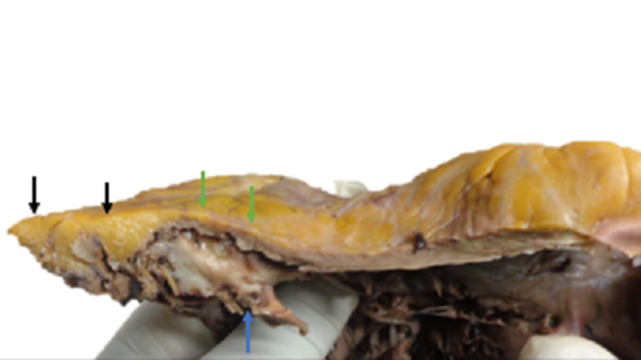
the right ventricular free wall showing ventricular wall thinning (green arrows) with marked fibrofatty replacement of the myocardium at the apex (black arrows), the papillary muscle (blue arrow) is also visible

However, the urea and creatinine levels remained elevated and his consciousness diminished (Glasgow coma scale -7/15). He was again dialysed, re-evaluated and had 3 more sessions of dialysis. In addition, he was transfused with packed red blood cells on 3 different occasions. The patient´s clinical state progressively declined as evidenced by the persistent respiratory distress and diminished consciousness. He died on the 8^th^ week of admission. An autopsy was performed upon the request of the family. External examination at autopsy revealed remarkable bilateral bony swelling at the knee joints and bilateral lower limb oedema. Internal examination revealed an enlarged heart weighing 600 gm. There was panvalvular dilatation with the tricuspid, pulmonary, mitral and aortic valves having circumferences of 13.0 cm, 9.0 cm, 9.5 cm, and 11.5 cm, respectively. The left and right ventricular wall thickness measured 2.0 cm and 0.6 cm, respectively. However, at the distal third of the right ventricular free wall, that is, at the apical region, there was thinning of the wall (0.2 cm) with subtotal fibrofatty replacement of the myocardium (Figure 1). One of the papillary muscles on the left side had an external diameter of 2.0 cm. Other findings included bilateral pulmonary oedema, hepatomegaly, chronic passive hepatic congestion, multiple simple renal cysts with focal benign papillary cortical adenoma, benign prostatic hyperplasia, complicated abdominal aortic atherosclerosis and presence of many corpora amylacea in the cerebral cortex.

**Figure 2:**
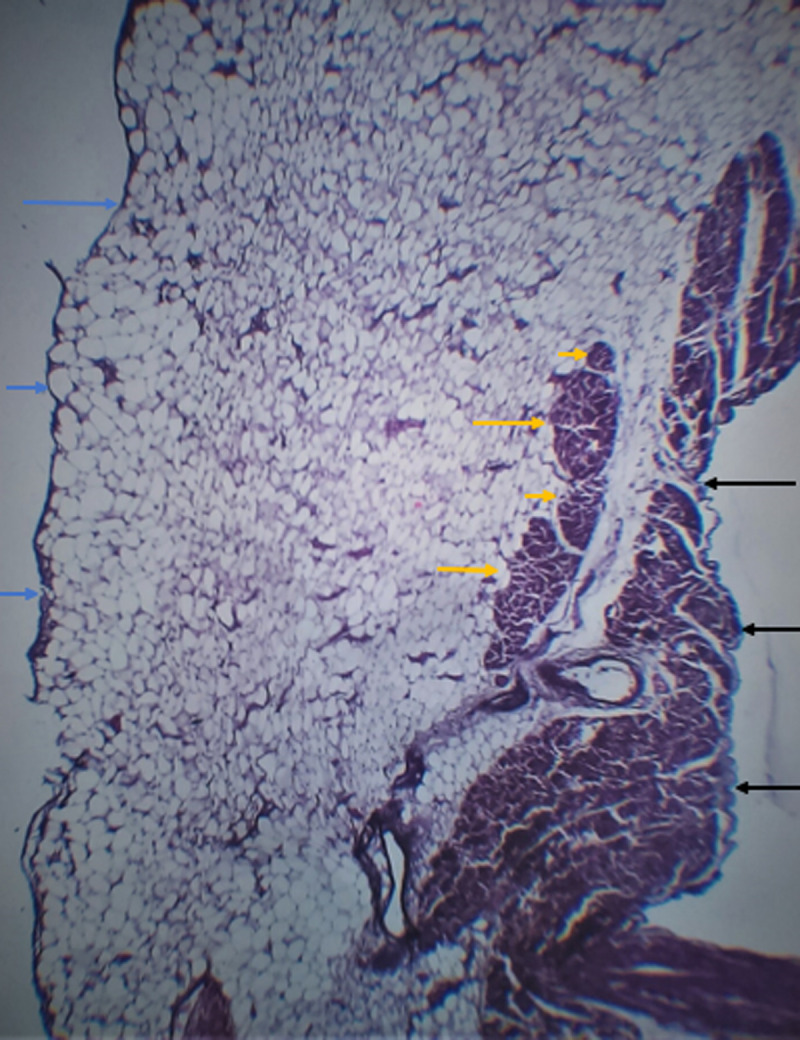
histological section shows a focal transmural replacement of cardiac myocytes by fibroadipose tissue extending from the epicardium (blue arrows) to the endocardial surface (black arrows), an island of atrophic myocytes is seen floating in the fibroadipose matrix (yellow arrows), H&E X40

**Figure 3:**
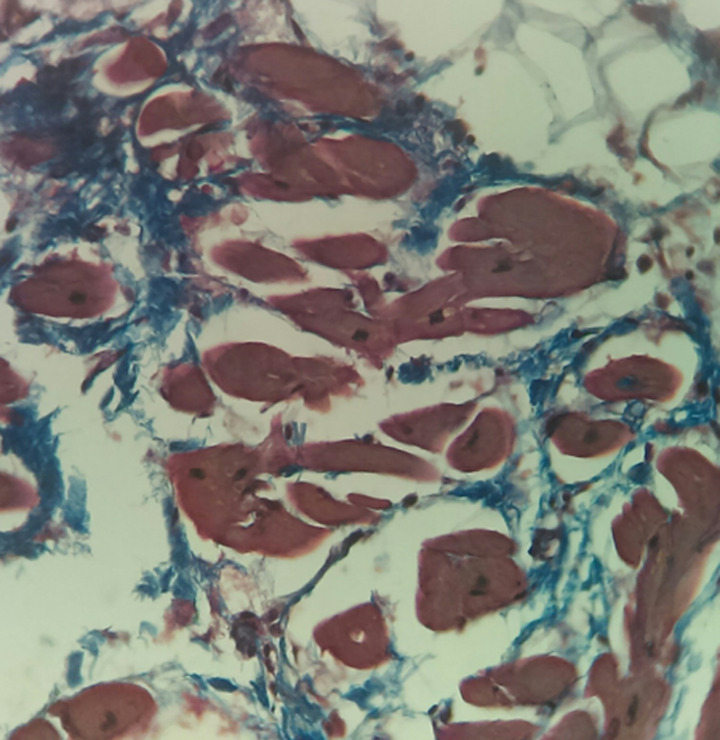
Masson trichrome - stained section of the heart highlighting the fibrous tissue (blue strands), X400

**Figure 4:**
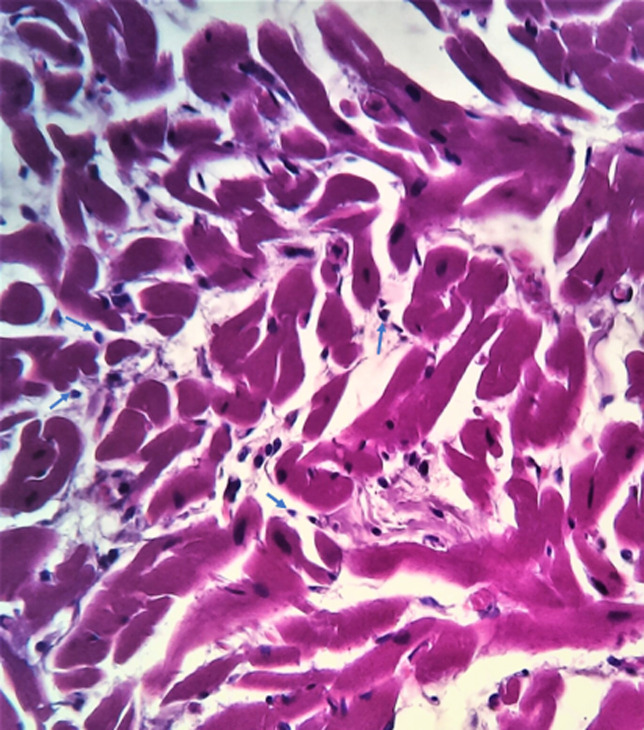
histological section shows myocyte atrophy with mild lymphocyte infiltrate (blue arrows), H&E X400

Microscopic examination of the sections of the heart taken from the apical region of the right ventricular wall revealed a transmural replacement of cardiac myocytes by fibroadipose tissue extending inwards, in the most parts, from the epicardium to the endocardial surface. Consequently, the myocardium showed at least 80-90% fat in-growth with focal transmural involvement (Figure 2). A Masson trichrome stain was done to highlight the fibrous tissue (Figure 3). Mild lymphocytic infiltrates were observed in parts (Figure 4). Sections taken from other parts of the right and left ventricular wall showed myocyte hypertrophy. The coronary vessels showed very mild eccentric atherosclerotic changes. Genetic studies were not done due to the unavailability of necessary facilities. Death was attributed to congestive cardiac failure due to hypertensive heart disease, with the right ventricular dysplasia being a significant and independent cause. The manner of death was concluded as natural.

## Discussion

The term right ventricular dysplasia (RVD) was coined by Fontaine in 1977 to describe a small group of patients who suffered from a strange heart disease characterised by ventricular tachycardia that originated from the right ventricle rather than from the usual left ventricular scar [8]. The more popular term “arrythmogenic” RVD (ARVD) was proposed because arrhythmia is the most frequent manifestation of this disease [9]. In the ensuing discussion, RVD and ARVD are used interchangeably. Thiene *et al*. in 1988, studied 60 hearts of young adults who had died from sudden cardiac death and observed RVD-like pathologies in 12 of them. They concluded that these individuals died from a cardiac muscle pathology of unknown aetiology, and named it right ventricular cardiomyopathy (RVC) [3]. In a later publication by this same group of researchers in 1996, the term arrhythmogenic right ventricular cardiomyopathy (ARVC) was introduced. That same year, the World Health Organisation (WHO) and the International Society and Federation of Cardiology (ISFC) task force on definition and classification of cardiomyopathies adopted the designation, ARVC, as the more appropriate name [10]. In other words, WHO and ISFC formally recognised ARVD as a cardiomyopathy and not a dysplasia; this partly de-emphasises suggestions of a congenital or growth abnormality. The ISFC task force went ahead to include ARVC as one of the five morphological and functional phenotypes of cardiomyopathies; the other four being dilated cardiomyopathy (DCM), hypertrophic cardiomyopathy (HCM), restrictive cardiomyopathy (RCM) and unclassified cardiomyopathy.

This development polarised the name of this disease entity amongst researchers: proponents of the “cardiomyopathy” terminology believe the disease to be an acquired condition, whereas, proponents of the “dysplasia” nomenclature believe that it is a developmental condition. Interestingly, Fontaine, who was the first to describe and name the disease, warned that “the term ‘cardiomyopathy´ may include too many different subtypes of diseased hearts with ARVD-like pathologies and render the search for a unified pathogenic mechanism and therapy very difficult, if not impossible” [11]. Right ventricular dysplasia presents classically with palpitations, syncope, or even sudden death [2]. The latter can be the first presenting symptom, particularly in young people under the age of 35 years and in athletes [3,4]. The incidence RVD is unknown; however, a prevalence of 1: 1000, with male predominance has been reported [12]. Right ventricular dysplasia is familial with an autosomal dominant mode of inheritance [13]; its aetiopathogenesis is associated with mutation in the genes encoding any of the five major components of the cardiac desmosomes. These genes are: *PKP2* (encoding plakophilin-2), *DSG2* (encoding desmoglein-2), *DSP* (encoding desmoplakin), *DSC2* (encoding desmocollin-2) and JUP (encoding junctional plakoglobin) [14].

Two pathological types have been described; the first is characterised solely by fat replacement of the right ventricle (fatty variant) while the second is characterised by fat infiltration and scarring of the right ventricle (fibrofatty variant). However, there is a controversy as to whether these types represent a spectrum of the same disease or whether they are different disease entities [15]. More controversies exist as regards the significance of myocardial fat infiltration. The fact that fat is present in the myocardium of normal hearts has posed a diagnostic challenge for anatomic/forensic pathologists at autopsy [16]. On the one hand, there is a tendency for the anatomic/forensic pathologists to regard intramyocardial fat as being physiological and underdiagnose RVD; or as pathological and over diagnose RVD. On the other hand, these features may as well go completely unnoticed. In other words, making the autopsy diagnosis of RVD could be problematic. The mere presence of adipose tissue, macroscopically, in the wall of the right ventricle is in itself insufficient to make the diagnosis; microscopic evaluation is pertinent. The microscopic examination of the retained sections revealed sheets of adipocytes interspersed by fibrous tissue strands. The latter is a more reliable feature for diagnosis [15,16]. The occurrence of myocyte atrophy with or without necrosis as well as the presence of lymphocyte infiltrates have also been reported [17,18]. Myocyte atrophy with mild lymphocyte infiltrate was observed in the index case (Figure 4). The fibrofatty infiltration occurs in the area of the heart referred to as the “triangle of dysplasia” [19]. The sides of the triangle are represented by the right ventricular outflow tract, the apex and the pulmonary infundibulum. In the present case, the fatty infiltration was in the apical region.

Another very important diagnostic feature, which has been reported in all cases, of RVD, is right ventricular (RV) wall thinning with or without aneurysmal dilatation. In the index case, the right ventricular wall thickness at the apical region measured 0.2 cm (reference interval = 0.3-0.5cm). Sometimes, there may be an isolated left ventricular or a biventricular involvement and where this is the case, the term arrhythmogenic cardiomyopathy (ACM) is employed [20]. To date, the growing body of literature on ARVD recognise RV wall thinning with fibrofatty replacement of the myocardium as the most consistent gross features, while myocyte atrophy with fat infiltration and fibrosis as the most reliable microscopic features.

## Conclusion

Considering the potentially fatal outcome of RVD, a correct diagnosis made at autopsy would immediately initiate the screening cascade of the deceased´s relatives.
